# CircHivep2 contributes to microglia activation and inflammation via miR‐181a‐5p/SOCS2 signalling in mice with kainic acid‐induced epileptic seizures

**DOI:** 10.1111/jcmm.15894

**Published:** 2020-10-01

**Authors:** Gao Xiaoying, Mian Guo, Liu Jie, Zhu Yanmei, Cui Ying, Shu Shengjie, Gou Haiyan, Sun Feixiang, Qi Sihua, Sun Jiahang

**Affiliations:** ^1^ Department of Anesthesiology The Fourth Affiliated Hospital of Harbin Medical University Harbin China; ^2^ Department of Neurosurgery The 2nd Affiliated Hospital of Harbin Medical University Harbin China; ^3^ Department of Neurology The 2nd Affiliated Hospital of Harbin Medical University Harbin China; ^4^ Department of Radiology The 2nd Affiliated Hospital of Harbin Medical University Harbin China; ^5^ Department of Imageology The 2nd Affiliated Hospital of Harbin Medical University Harbin China

**Keywords:** circular RNAs, epilepsy, inflammatory response, microglia activation, miR‐181a‐5p

## Abstract

Epilepsy is a chronic brain disease characterized by recurrent seizures. Circular RNA (circRNA) is a novel family of endogenous non‐coding RNAs that have been proposed to regulate gene expression. However, there is a lack of data on the role of circRNA in epilepsy. In this study, the circRNA profiles were evaluated by microarray analysis. In total, 627 circRNAs were up‐regulated, whereas 892 were down‐regulated in the hippocampus in mice with kainic acid (KA)‐induced epileptic seizures compared with control. The expression of circHivep2 was significantly down‐regulated in hippocampus tissues of mice with KA‐induced epileptic seizures and BV‐2 microglia cells upon KA treatment. Bioinformatics analysis predicted that circHivep2 interacts with miR‐181a‐5p to regulate *SOCS2* expression, which was validated using a dual‐luciferase reporter assay. Moreover, overexpression of circHivep2 significantly inhibited KA‐induced microglial activation and the expression of inflammatory factors in vitro, which was blocked by miR‐181a‐5p, whereas circHivep2 knockdown further induced microglia cell activation and the release of pro‐inflammatory proteins in BV‐2 microglia cells after KA treatment. The application of circHivep2+ exosomes derived from adipose‐derived stem cells (ADSCs) exerted significant beneficial effects on the behavioural seizure scores of mice with KA‐induced epilepsy compared to control exosomes. The circHivep2+ exosomes also inhibited microglial activation, the expression of inflammatory factors, and the miR‐181a‐5p/SOCS2 axis in vivo. Our results suggest that circHivep2 regulates microglia activation in the progression of epilepsy by interfering with miR‐181a‐5p to promote *SOCS2* expression, indicating that circHivep2 may serve as a therapeutic tool to prevent the development of epilepsy.

## 1 INTRODUCTION

Epilepsy is a chronic neurological disorder that results from the activation of an aberrant neural network.^1^, ^2^, ^3^ Many forms and causes of the condition exist but all result in a common reactive process involving the synchronous hyperexcitation of neurons, which ultimately gives rise to spontaneous and recurrent seizures.^4^, ^5^, ^6^ Inflammation is integral to the hyperexcitation that originates in affected brain tissue and a number of inflammatory processes are associated with epileptogenesis, such as signalling associated with pro‐inflammatory proteins and interactions between neurons and microglia.^7^, ^8^ Many antiepileptic drugs are currently in use to alleviate symptoms but they are associated with severe adverse effects that limit their use.^9^, ^10^ Therefore, a greater understanding of the regulatory and molecular processes that govern the events leading to epileptic seizures may lead to more precise and relevant therapeutic targets with fewer adverse effects.

Several studies have investigated whether epigenetic processes, such as those involving DNA methylation or non‐coding RNA, may be associated with epileptogenesis.^11^, ^12^, ^13^ Moreover, it has been suggested that the future of epileptic management could involve microRNA (miRNA)‐targeted therapies following the revelation by genome‐wide analysis that many genes are differentially regulated during seizures.^14^, ^15^ It is becoming evident that miRNAs are involved in the process and maintenance of the epileptic state through the regulation of inflammation, stress signalling and neuronal excitation.^16^ For instance, the involvement of miR‐34a has been identified in seizure‐induced neuronal death whereas down‐regulating the expression of miR‐134 reduced the occurrence of epileptic seizures and their associated damage.^17^ The identification of more epilepsy‐regulated miRNAs would be useful in the future management of the disease. Circular RNAs (circRNAs) are endogenous non‐coding RNAs that have been proposed to regulate gene expression predominantly through the sequestration of target miRNAs.^18^, ^19^ A notable number of circRNA are present in the brain compared with other mammalian tissue but whether they function in epilepsy is unclear.^13^, ^20^, ^21^

One objective of the present study was to identify circRNA that may be differentially expressed during seizures and to identify their targets. To this end, we constructed a circRNA expression profile from the hippocampus of mice with kainic acid (KA)‐induced status epilepticus (SE).^22^ Of the circRNA that were differentially expressed in the hippocampus of mice induced with SE, we identified that circRNA‐0000146 (chr10: 13724006‐13786490) was consistently down‐regulated. We found that circRNA‐0000146 was derived from *Hivep2* and therefore named it CircHivep2. In humans, *HIVEP2* encodes human immunodeficiency virus type I enhancer‐binding protein 2, which is a C2H2 zinc finger protein also known as the transcription factor ZAS2/MIBP1 and related to neurological disorders characterized by intellectual disability and physical characteristics.^23^, ^24^, ^25^ HIVEP2 has been associated with numerous regulatory pathways, including cMyc and NF‐κB,^26^, ^27^ and is involved in cellular immunity and development processes.^25^, ^28^

Therefore, in the present study, we have identified the circRNA that are differentially expressed in a KA‐induced model of SE in mice and have further assessed CircHivep2 because it may play a pivotal role in the regulation of epileptic seizures. We have carried out expression differentiation experiments using CircHivep2 and identified its key interaction partners. One of the major problems in using non‐coding RNA in the therapy of neurological diseases is the challenge of traversing the blood‐brain barrier. Adipose‐derived stem cells (ADSCs) are an important source of exosomes that are involved in the regeneration and repair of the nervous system.^29^ More importantly, exosomes can traverse the blood‐brain barrier to deliver miRNAs directly to the brain and have been proposed as a potential delivery system to treat epilepsy.^30^, ^31^ In this study, we exploit the use of exosomes from ADSCs to deliver CircHivep2 into a mouse model of SE. We have also examined the influence this has on inflammatory processes by measuring the level of pro‐inflammatory cytokines released from glia activated by the overexpression of CircHivep2 in hippocampus tissue. This study aims to advance our knowledge on the regulation of SE by circRNA.

## 2 MATERIALS AND METHODS

### 2.1 Animals, epilepsy model and treatments

Adult male C57BL/6 mice (8‐10 weeks of age) were purchased from the Laboratory Animal Center of Harbin Medical University and maintained in standard conditions with food and water ad libitum. All animal studies were approved by the Institutional Animal Care and Use Committee of the Fourth Affiliated Hospital of Harbin Medical University (reference number: KYZ18‐108; Harbin, Heilongjiang, China).

**Figure 1:**
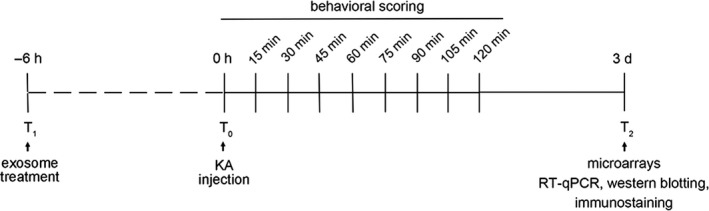
Overview of the time scale for treatment and seizure induction, behavioural scoring. T_0_, time point of kainic acid (KA) injection; T_1_, time point for the acute treatment of PBS, exosomes (Exo), control vector‐Exo or pCD‐circHivep2‐Exo (n = 10/group). For time‐course analysis of KA‐induced epilepsy, a duration of 120 min was chosen. T_2_, time point for microarrays, RT‐qPCR, Western blotting and immunostaining in hippocampus tissues of mice with KA‐induced epilepsy, at 3 d after seizures commenced

An induced SE model was established by an i.p. injection of 30 mg/kg of KA (Sigma‐Aldrich) in 0.9% saline at a volume of 10 mL/kg body weight. The mouse behaviour was observed for 2 hours thereafter and scored every 15 minutes (Figure 1). The occurrence of seizures and their severity were blind‐scored by two investigators, using the following classification stages^32^: 0, no response; 1, immobile and staring; 2, forelimb and/or tail extension, rigid posture; 3, repetitive movements, head bobbing; 4, rearing and falling; 5, continuous rearing and falling: 6, severe tonic‐clonic seizures; 7, death. The mice progressed at least to stage 3 and were killed 3 days after seizures commenced. Controls were injected with a vehicle (0.9% saline, 10 mL/kg body weight).

For exosome treatment (Figure 1), 6 hours before SE was induced by injection of KA, mice (n = 10/group) received an intracerebroventricular injection of PBS or ADSC‐derived exosomes with or without circHivep2 overexpression or control vector plasmid at 10 nmol/kg as previously described.^33^ Briefly, the mice exosomes were injected with a 5‐μL Hamilton syringe in the right lateral cerebral ventricle 0.5 mm posterior to the bregma, 1 mm lateral to the midsagittal suture, and 2‐2.5 mm below the dura mater. At the times indicated, mice were killed by decapitation under light diethyl‐ether anaesthesia and brains were removed rapidly. The hippocampi were dissected and immediately stored at −80°C.

### 2.2 Expression profile analysis of circRNAs

Total RNAs were first extracted from hippocampi using Trizol reagent (Invitrogen) following the manufacturer's instructions. Total RNA was purified using an RNeasy Mini Kit (Qiagen) and quantified with a Nanodrop ND‐1000 (Thermo Fisher Scientific). After the quality of the RNA was checked (Agilent 2100 Bioanalyzer, Agilent Technologies), the samples were prepared for microarray hybridization (Arraystar). To enrich circRNA and remove linear RNA, RNA was treated with RNase R (Epicenter). The samples enriched with circRNA were amplified with a random priming method and transcribed into fluorescent cRNA (Arraystar Super RNA Labeling Kit) and hybridized onto an array of mouse circRNA (8 × 15K, Arraystar Mouse circRNA Array V2) following manufacturer's instructions. Finally, the slides were washed and scanned (Agilent Scanner G2505C, Agilent Technologies) and the acquired array images were analysed. Quantile normalization and subsequent data processing were performed using the R software limma package. Differentially expressed circRNAs were selected according to *P*‐value (<.05) and fold change cutoff (≥2). Hierarchical clustering was performed to identify distinguishable circRNAs expression pattern among samples.

### 2.3 Quantitative real‐time PCR

Total RNA and miRNAs were extracted from the cultured cells and tissues using Trizol reagent and a mirVana miRNA isolation kit (Ambion) according to the manufacturer's instructions. Subsequently, cDNA was synthesized and amplified by RT‐qPCR based on the TaqMan method on an ABI PRISM 7500 Sequence Detection System (Life Technologies). U6 snRNA was used as the internal control for miRNAs, and circRNA levels were normalized to GAPDH. Relative expression levels of the genes were calculated using the 2^−ΔΔCt^ method.

### 2.4 Cell culture and transfection

BV‐2 cells, an immortalized murine microglial cell line, were obtained from the Cell Bank of Chinese Academy of Sciences (Shanghai, China) and cultured in DMEM supplemented with 10% foetal bovine serum (FBS) and 1% penicillin/streptomycin (Gibco) at 37°C in a humidified atmosphere containing 95% air and 5% CO_2_. The cells were then sub‐cultured into 24‐well plates and maintained until subconfluence.

The full‐length cDNA of circHivep2 was inserted into pcDNA3.1 and the mock plasmid without the circHivep2 cDNA served as the control. Small interfering RNAs (siRNAs) targeting mouse circHivep2 (si‐circHivep2) or mouse TDP‐43 (siTDP‐43) and non‐specific negative control oligos (si‐NC or control siRNA), mouse miR‐181a‐5p‐mimics and the corresponding negative control mimic (miR‐NC) were obtained from Gene Pharma Co. Ltd. Transfection was performed using Lipofectamine 3000 (Invitrogen) following the manufacturer's instructions. The effects of knockdown or overexpression were examined by RT‐qPCR using RNA extracted 48 hours after transfection.

For treatment, BV‐2 cells were pre‐incubated with control vector or circHivep2 overexpression plasmid, si‐NC or si‐ circHivep2, miR‐NC or miR‐181a‐5p for 30 minutes and then treated with or without KA (50 μmol/L) for 2 or 12 hours.

### 2.5 Western blot analysis

Cells were lysed and the protein concentration was measured using a bicinchoninic acid (Thermo Fisher Scientific). Protein samples (50 μg) were separated by using 10% SDS‐polyacrylamide electrophoresis and then transferred to nitrocellulose membranes (Sigma‐Aldrich). After blocking the membranes in 5% non‐fat milk at room temperature for 1 hour they were incubated at 4°C overnight with primary antibodies: anti‐Iba1, anti‐TNF‐α, anti‐IL‐1β, anti‐SOCS2, anti‐TDP‐43, anti‐CD9, anti‐CD63, anti‐TSG101 and β‐actin (Abcam) followed by secondary antibodies (1:5000 dilutions, Jackson ImmunoResearch) for 1 hour. The signals were then visualized with an Odyssey Infrared Imaging System (LI‐COR Biosciences) after three washes.

### 2.6 Biotinylated RNA probe pull‐down assay

After washing in ice‐cold PBS BV‐2 microglia cells (1 × 10^7^) were lysed and incubated at room temperature for 2 hours with a negative control probe or a circHivep2 or miR‐181a‐5p probe labelled with high‐affinity biotin. Streptavidin magnetic beads (50 μL, Thermo Fisher Scientific) were added to the mixture, which was incubated for a further 1 hour. The RNA was purified and then analysed by qRT‐PCR. The protein reserved in circHivep2 RNA was analysed by Western blotting using anti‐TDP‐43 antibodies.

### 2.7 Fluorescence in situ hybridization (FISH)

To identify the localization of miR‐181a‐5p and circHivep2 in BV‐2 microglia cells, in situ hybridization was conducted using probes specific for both RNA molecules: cy3‐labelled probes for miR‐181a‐5p or miR‐NC and fluorescein isothiocyanate probes for circHivep2 sequence or negative control (circ‐control). Cell nuclei were counter‐stained with 4,6‐diamidino‐2‐phenylindole (DAPI), and images were captured using an Olympus BX53 Microscope (Olympus).

### 2.8 Luciferase reporter assay

Constructs containing wild‐type or mutant circHivep2‐miR‐181a‐5p and SOCS2‐miR‐181a‐5p were subcloned with a luciferase gene using a psiCHECK‐2 vector (Promega Corporation) or a pmirGLO vector (Promega Corporation), respectively. Lipofectamine 2000 (Invitrogen) was used to transfect cells according to manufacturer's instructions, 40 ng of luciferase reporter vectors and 10 pmol miR‐181a‐5p mimics/NC were transfected into 293 cells for 24 hours. Thereafter, firefly and Renilla luciferase activities were measured continuously using a dual‐luciferase reporter assay system (Promega Corporation). Finally, firefly to Renilla luciferase ratios was calculated for each well, and each measurement was repeated three times in three independent experiments.

For pri‐miRNA processing assays, fragments of pri‐mir‐181a‐5p containing the hairpin and 100 bp flanking sequence were amplified from genomic DNA. The PCR products were digested and inserted into the pmirGLO vector downstream to firefly luciferase reporter. Cropping of the hairpin stem‐loop of the inserts results in destabilization of the firefly reporter leading to a decrease in firefly luminescence. Dual‐luciferase reporters with pri‐mir‐181a‐5p were transfected in BV‐2 microglia cells using Lipofectamine 2000 (Invitrogen).

### 2.9 Exosomes preparation and identification

Exosomes were prepared from ADSCs as previously described.^34^ The circHivep2 overexpression or control vector plasmids were transfected into ADSC cells using Lipofectamine 3000 (Invitrogen) according to the manufacturer's instructions. ADSCs or ADSCs transfected with circHivep2 overexpression or control vector plasmids were used to prepare exosomes (Exo), control vector‐Exo and pCDcircHivep2‐Exo, respectively. ADSCs were grown to 80% confluence and washed three times in PBS and cultured in exosome‐depleted media containing FBS (Sigma‐Aldrich) for 48 hours. The medium was then passed through a 0.22 μm filter (BD Falcon), and exosomes were extracted using ExoQuick Exosome Precipitation Solution (System Biosciences) by centrifugation for 30 minutes at 250 *g* and then 5 minutes at 1000 *g* after an overnight incubation at 4°C. The exosomes were resuspended in 200 μL media. Protein markers, CD9, CD63, TSG101 were determined by immunoblotting. The BCA protein assay kit was used to quantify the exosomes. The numbers and sizes of exosomes were directly measured using the Nanosight NS 300 system (NanoSight Technology).

### 2.10 Immunofluorescence staining

Microglial cells grown on cover slides were washed with PBS and then fixed for 30 minutes with paraformaldehyde (4% in PBS). After cells were permeabilized with 0.3% Triton X‐100 for 15 minutes, they were blocked with normal goat serum for 30 minutes before overnight incubation with mouse primary antibody specific to Iba1 (1:100 dilution, Wako) at 4°C. They were finally washed in PBS and incubated with Alexa Fluor 594‐conjugated goat anti‐mouse secondary antibody (1:500; Life Technology) for 2 hours at room temperature. After washing three times with PBS, cells were stained with DAPI and then observed for positive indication of microglia morphology under an Olympus FV10‐ASW confocal microscope (Olympus).

Brain sections used for immunofluorescence were incubated overnight with one pair of the following antibodies: anti‐Iba1 (1:500; Abcam), anti‐Interleukin 1 beta (IL‐1β; 1:500; Santa Cruz) and antitumor necrosis factor‐alpha (TNF‐α; 1:1000; Santa Cruz). The sections were then incubated with Texas Red‐conjugated IgG (1:400; Vector Laboratories) and fluorescein isothiocyanate‐conjugated IgG (1:200; Jackson ImmunoResearch), and mounted with Vectashield mounting medium (Vector Laboratories). The stained sections were examined under a microscope (Olympus). The expression of circHivep2 was determined by fluorescent in situ hybridization (FISH) using a double FAM‐labelled Detection Probe (Qiagen) in combination with immunohistochemical labelling for Iba1 + microglia colocalization.

### 2.11 Statistical analysis

All data are presented as the mean ± standard deviation (SD). Statistical significance was determined by the Student's *t* test or one‐way analysis of variance (ANOVA) followed by Tukey's post hoc test (GraphPad Prism). *P*‐values < .05 were considered statistically significant. All experiments were performed in triplicate at least.

## 3 RESULTS

### 3.1 Expression profiles of circRNAs in the hippocampus of a mouse model during KA‐induced epilepsy

**Figure 2:**
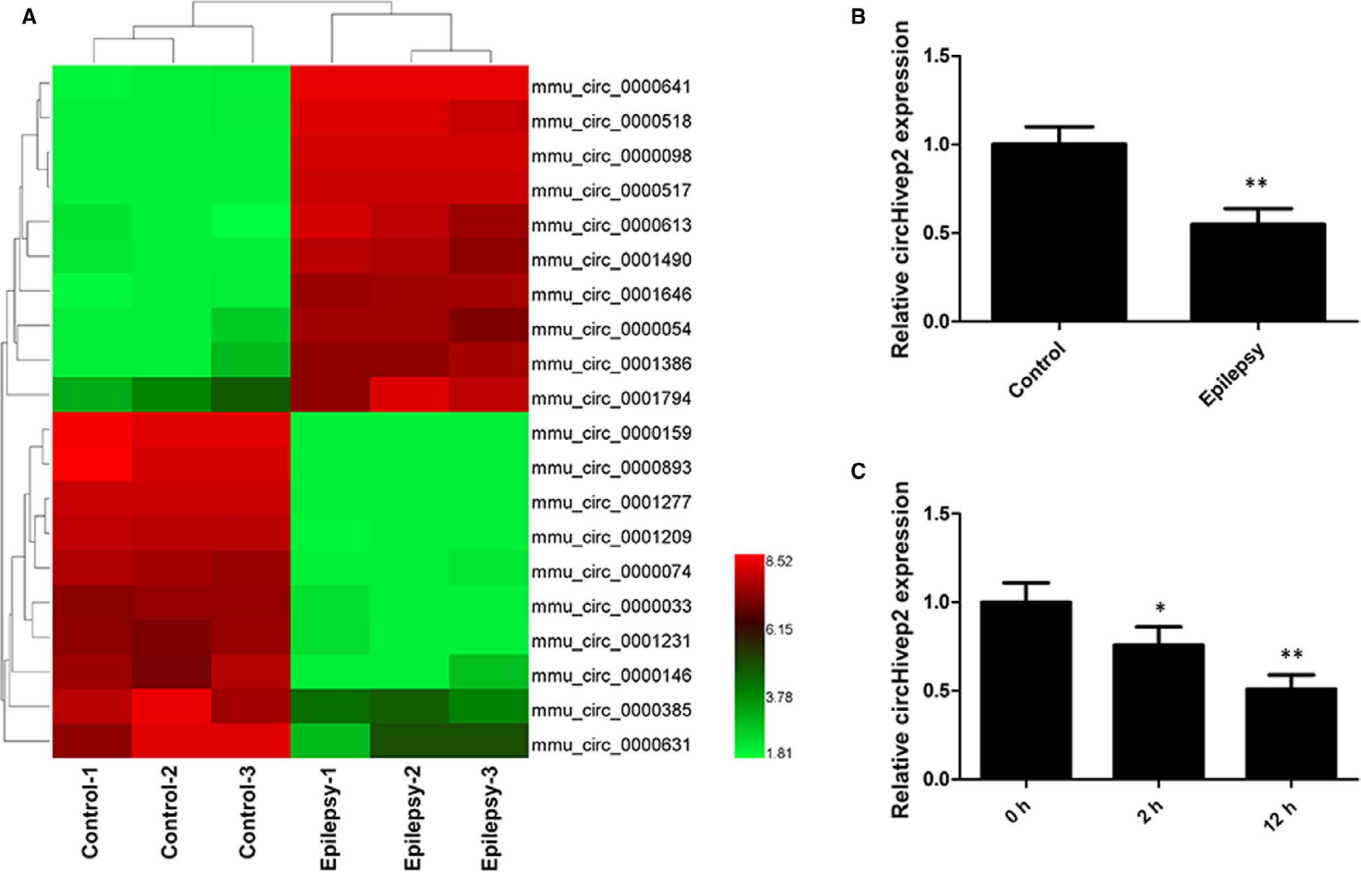
Deregulated circular RNAs (circRNAs) in the hippocampus of a mouse model of kainic acid (KA)‐induced epilepsy. The mice progressed at least to stage 3 and were killed 3 d after seizures commenced. The hippocampus tissues were collected and the circRNA profiles were assessed. A, A heatmap showed 20 aberrantly expressed circRNAs, including 10 up‐regulated and 10 down‐regulated circRNAs in mice hippocampus with KA‐induced epilepsy compared with control samples (n = 3/group). Red represents high expression, whereas green represents low expression. B, RT‐qPCR verification of the expression of circRNA‐0000146 (circHivep2) in hippocampus tissues of mice with KA‐induced epilepsy as compared with controls (n = 10/group). C, RT‐qPCR verification of the expression of circHivep2 in BV‐2 microglial cells after KA treatments for 2 or 12 h

We have used a circRNA microarray technique to evaluate the circRNA profiles in the hippocampus of mice with KA‐induced epileptic seizures and compared these profiles with controls. In response to KA‐induced epileptic seizures 627 circRNAs were up‐regulated, whereas 892 circRNAs were down‐regulated based on log2 (fold changes) ≥2, *P* < .05 and false discovery rate (FDR) <0.05. The top 10 up‐regulated and 10 down‐regulated circRNAs in the epilepsy group are listed in Table 1. Figure 1 shows a heatmap of 20 circRNAs, 10 up‐regulated and 10 down‐regulated, that exhibited the greatest changes in expression during epilepsy (Figure 2A). The expression levels of these circRNA were validated by RT‐qPCR. There was a general consistency between the high‐throughput data and qPCR results. In particular, we observed that the expression of circRNA‐0000146 (chr10: 13724006‐13786490) was significantly reduced in hippocampus tissues of mice compared with controls (Figure 2B, *P* < .01). By browsing the mouse reference genome (NCBI37/mm9), we identified that circ_0000146 is derived from Hivep2, which is located on chromosome 10, and thus we named it circHivep2. We selected circHivep2 for further assessment and found that its expression decreased significantly in BV‐2 microglia cells from 2 to 12 hours after the addition of KA when compared to the control group (Figure 2C). Overall, we have established a profile of 1519 circRNA that are differentially expressed in the hippocampus during KA‐induced epileptic seizures in mice. Of these differentially expressed circRNAs, we selected circHiveP2 for further investigation because it may be involved in pathways associated with epileptic seizures.

**Table 1:** Top 20 differently expressed circRNAs in microarray analysis

circRNA	Gene symbol	*P*‐value	FDR	Fold change	Rengulation
mmu_circ_0000641	Nell2	.000107	0.0883042	6.567551	Up
mmu_circ_0000518	Cacna2d3	.000201	0.1031693	6.453341	Up
mmu_circ_0000098	Ralgps2	.002095	0.2937934	6.329306	Up
mmu_circ_0000517	Erc2	.012539	0.1863075	6.19356	Up
mmu_circ_0000613	Ptk2	.008476	0.1511231	6.043781	Up
mmu_circ_0001490	Gmcl1	.023193	0.2056082	5.876587	Up
mmu_circ_0001646	Inpp5a	.028069	0.186424	5.743814	Up
mmu_circ_0000054	Acsl3	.017643	0.1785026	5.687446	Up
mmu_circ_0001386	Ep400	.015391	0.1695523	5.469712	Up
mmu_circ_0001794	Map2k1	.005184	0.1256518	4.127446	Up
mmu_circ_0000159	Gtf3c6	.000421	0.0562795	6.503082	Down
mmu_circ_0000893	Dcc	.000833	0.0971607	6.352845	Down
mmu_circ_0001277	Eya3	.00678	0.149682	6.185125	Down
mmu_circ_0001209	Ikbkap	.019354	0.1629713	5.995309	Down
mmu_circ_0000074	Ccnt2	.003802	0.0961156	5.776681	Down
mmu_circ_0000033	Gulp1	.014187	0.1236089	5.518901	Down
mmu_circ_0001231	BC057079	.002675	0.1110218	5.337383	Down
mmu_circ_0000146	Hivep2	.000942	0.0871523	5.204817	Down
mmu_circ_0000385	Nemf	.039146	0.2173156	3.600157	Down
mmu_circ_0000631	Atxn10	.006915	0.1596431	3.4641	Down

### 3.2 circHivep2 influences the levels of miR‐181a‐5p and *SOCS2* is a direct target of miR‐181a‐5p

**Figure 3:**
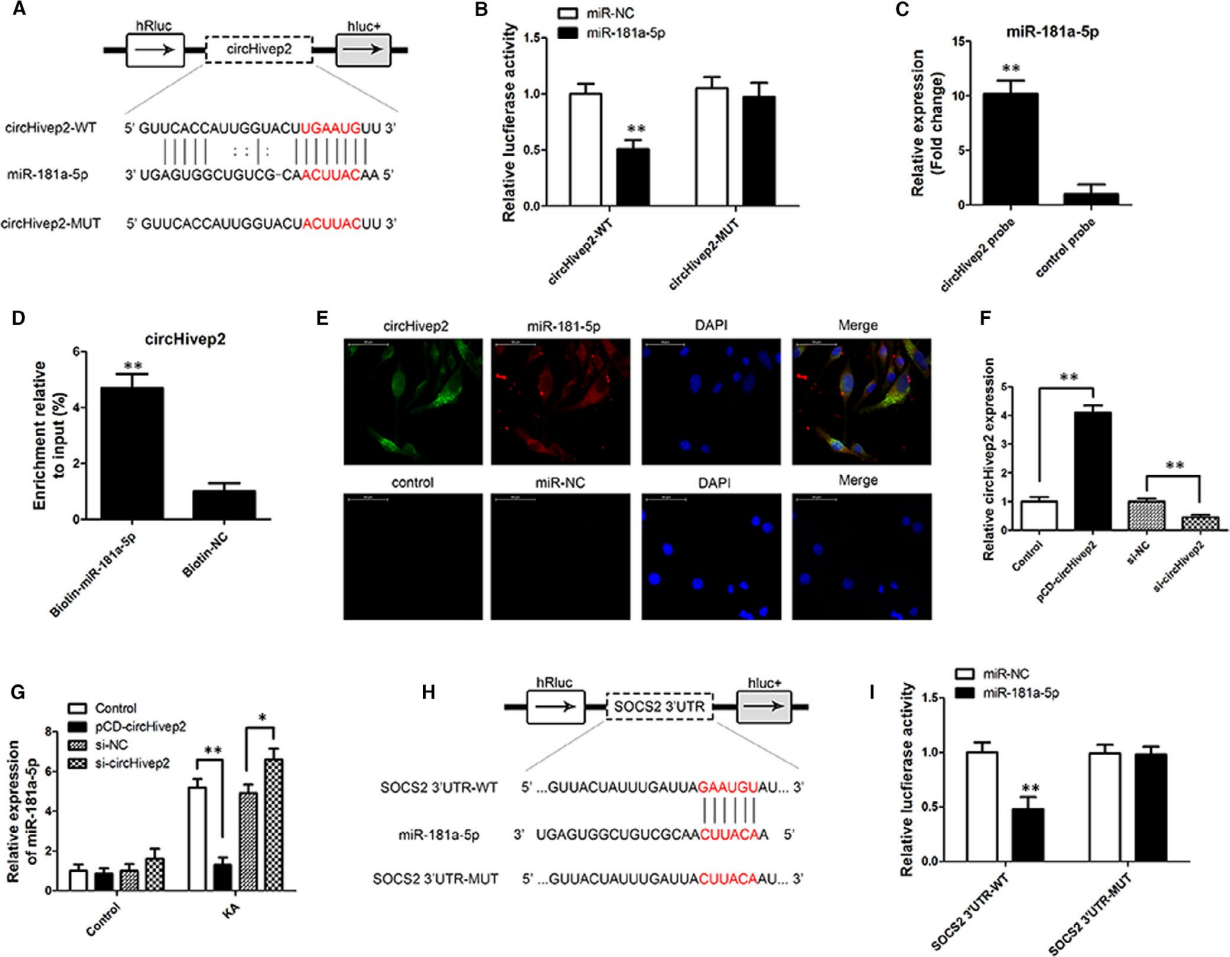
CircHivep2 acts as a sponge for miR‐181a‐5p, and *SOCS2* is a direct target of miR‐181a‐5p. A, Putative complementary sites within miR‐181a‐5p and circHivep2 were predicted by bioinformatics analysis. B, Dual‐luciferase reporter assays demonstrate that miR‐181a‐5p is a direct target of hsa_circ_0000146 (**P < .01, n = 3/group). C, miR‐181a‐5p was pulled down and enriched with a circHivep2 specific probe and then detected by qRT‐PCR (**P < .01, n = 3/group). D, RT‐PCR analysis of circHivep2 captured by biotin‐miR‐181a‐5p (n = 3/group). E, Colocalization of circHivep2 and miR‐181a‐5p in the cytoplasm by RNA FISH assay (magnification, 400×, n = 3/group). Nuclei are stained blue (DAPI), circHivep2 or control is stained green, and miR‐181a‐5p or miR‐NC is stained red. F, RT‐qPCR for circHivep2 expression in BV‐2 microglial cells treated with control vector or circHivep2 overexpression plasmid, si‐NC or si‐ circHivep2 (n = 3/group). G, The level of miR‐181a‐5p was determined by RT‐PCR in BV‐2 microglia cells with control vector or circHivep2 overexpression plasmid, si‐NC, or si‐ circHivep2 after KA treatment (n = 3/group). H, Predicted binding sites between *SOCS2* and miR‐181a‐5p as predicted by bioinformatics analysis. I, Luciferase reporter assay demonstrated miR‐181a‐5p mimics significantly decreased the luciferase activity of S*OCS2*‐wt in HEK293T cells (n = 3/group). Data are the means ± SD. *P < .05, **P < .01, Student's*t* test or two‐way ANOVA

To identify any potential interaction between circHivep2 and other microRNA, we queried two public databases, circBase (http://www.circbase.org) and StarBase, version 3.0 (http://starbase.sysu.edu.cn/), and found that circHivep2 could interact with miR‐181a‐5p. To verify this, we performed a dual‐luciferase reporter assay after mutating the potential miR‐181a‐5p binding site in circHivep2 (Figure 3A). The results indicated that miR‐181a‐5p decreased the luciferase activities of the wild‐type (WT) reporter for circHivep2 but not the activities of the mutant (MUT) reporter (*P* < .01) (Figure 3B), suggesting direct contact between circHivep2 and miR‐181a‐5p. In humans, the miR‐181 group of microRNAs are found to be expressed in the immune system, retina and the brain.^35^, ^36^ The overexpression of miR‐181a‐5p has been found to be involved in cell proliferation and differentiation, and it is up‐regulated in neurological conditions.^37^, ^38^ We assessed whether miR‐181a‐5p could interact directly with circHivep2 in vitro through the use of biotinylated pull‐down assays. Compared with the control group, more miR‐181a‐5p was detected in the circHivep2 probe‐captured fraction (Figure 3C). Additionally, higher enrichment of circHivep2 was observed in the biotin‐coupled miR‐181a‐5p groups than in the biotin‐coupled negative control (NC) groups (Figure 3D). The results of RNA pull‐down assays confirmed that circHivep2 can bind to miR‐181a‐5p. Moreover, an RNA FISH assay indicated that circHivep2 and miR‐181a‐5p were found to both localize in the cytoplasm of BV‐2 microglia cells (Figure 3E).

Experiments to access the differential expression of circHivep2 were also carried out in BV‐2 microglia cells (Figure 3F). Overexpression of circHivep2 in cells transfected with pCD‐circHivep2 resulted in significantly increased expression whereas down‐regulation of circHivep2 in cells transfected with si‐circHivep2 resulted in decreased expression. Levels of miR‐181a‐5p were measured under the same experimental conditions and with the addition of KA (Figure 3G). Over‐ and under‐regulation of circHivep2 did not have a significant impact on the level of miR‐181a‐5p under normal conditions but when cells were treated with KA the levels of miR‐181a‐5p increased significantly in control cells and reached the highest level in cells with circHivep2 expression down‐regulated. However, the level of miR‐181a‐5p expression was the lowest in cells overexpressing circHivep2. Indicating that KA induced the expression of miR‐181a‐5p but the presence of circHivep2 inhibited the induction of miR‐181a‐5p expression. Accumulating evidence has shown that post‐transcriptional regulation, rather than transcriptional regulation, is critical in determining the levels of mature miRNAs. Some studies have found that several RBPs, such as DDX1, TDP‐43, KSRP and SMAD4, are regulatory components that interact with miRNA processing machinery and guide the maturation of specific subsets of miRNAs.^39^, ^40^, ^41^, ^42^ It has also been demonstrated that TDP‐43 is pathologically accumulated in cytoplasmic aggregates in neurodegenerative disorders and regulates the activity of miRNAs biogenesis machinery, in particular, Drosha and Dicer.^39^, ^43^ Therefore, we have examined if TDP‐43 levels are altered in KA‐treated BV‐2 microglia cells. The results of Western blotting show that the protein level of TDP‐43 was increased by KA, the TDP‐43 protein level was greatly reduced both in untreated and in KA‐treated BV‐2 cells after TDP‐43 knockdown by siRNA (Figure S1A). The levels of miR‐181a‐5p were unaffected or slightly affected by siTDP‐43 in untreated cells; conversely, they all were significantly reduced in KA‐treated cells after TDP‐43 knockdown (Figure S1B). Meanwhile, knockdown of TDP‐43 impaired the microprocessor processing of pri‐miR‐181a‐5p expressed as increased firefly/renilla luminescence, while this effect was rescued by overexpression of circHivep2 and enhanced by si‐circHivep2 (Figure S1C). Next, we investigated the interaction between circHivep2 and TDP‐43. The expression circHivep2 and TDP‐43 were not regulated by each other (Figure S1D,E). However, RNA pull‐down followed by Western blot assay confirmed that circHivep2 did interact with TDP‐43 (Figure S1F). These results suggest that circHivep2 could block the interaction between pri‐miR‐181a‐5p and TDP‐43 and impair the microprocessor‐mediated biogenesis of miR‐181a‐5p.

The importance of circHivep2 and miR‐181a‐5p expression in microglia cells treated with KA led us to investigate the potential binding partners of miR‐181a‐5p. TargetScan (http://www.targetscan.org/vert_71/) allowed us to identify the gene suppressor of cytokine signalling 2 (*SOCS2*) as a possible target of miR‐181a‐5p. *SOCS2* encodes a protein that is believed to interact with the cytoplasmic domain of insulin‐like growth factor 1 receptor (IGF1R) and forms part of a pathway involved in IGF‐1 regulated growth control.^44^ A luciferase assay with a mutation in the putative miR‐181a‐5p binding site in *SOCS2* allowed us to confirm that *SOCS2* was a target of miR‐181a‐5p (Figure 2H,I). The miR‐181a‐5p mimics significantly decreased the luciferase activity of *SOCS2*‐wt in HEK293T cells. Overall, these results begin to suggest that the expression of miR‐181a‐5p is induced by KA which would then increase its ability to bind to its target gene *SOCS2*. Overexpression of circHivep2 seems to sequester or inhibit the expression of miR‐181a‐5p, thereby circHivep2 seems to negatively regulate miR‐181a‐5p.

### 3.3 circHivep2 regulates microglia activation via the miRNA‐181a‐5p/SOCS2 pathway in vitro

**Figure 4:**
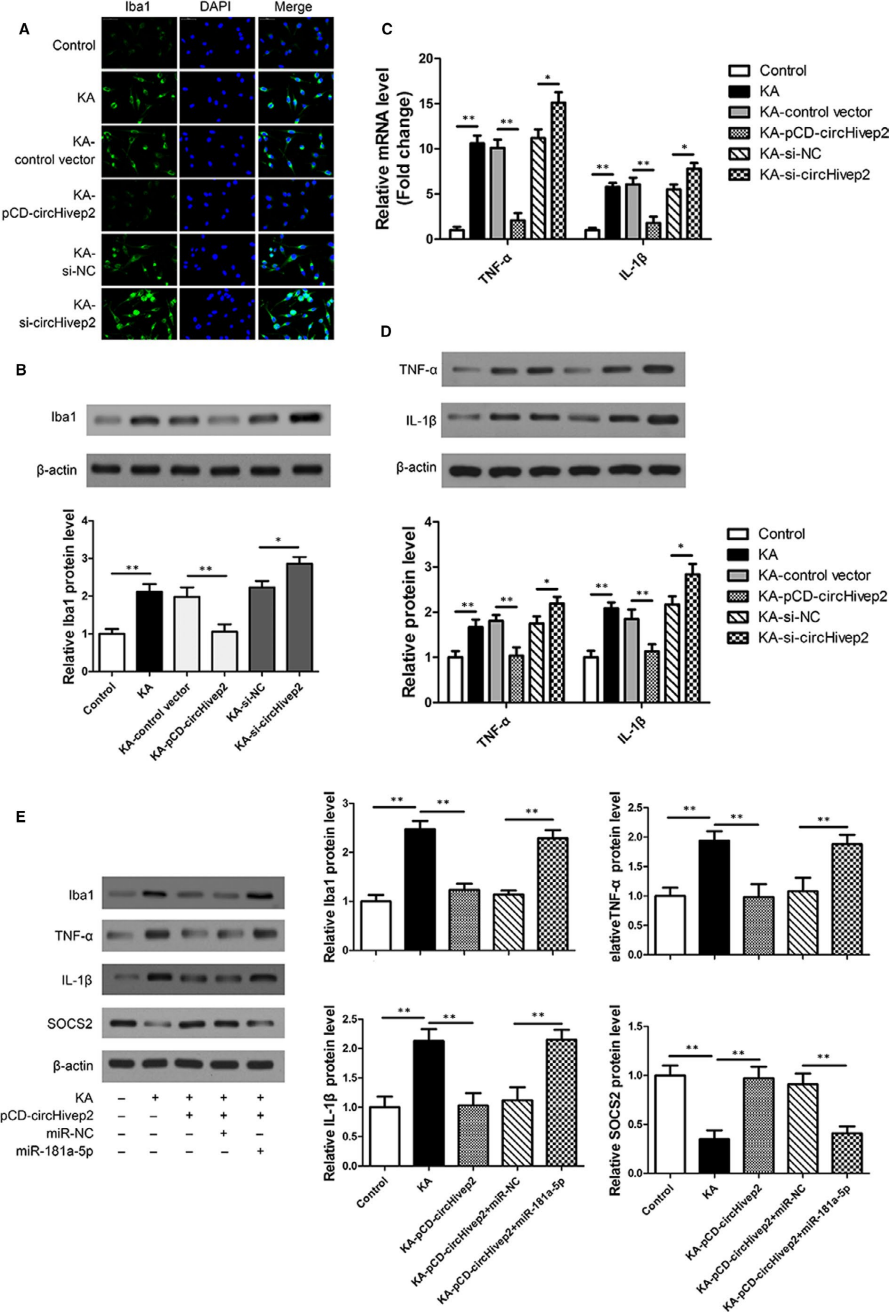
circHivep2 regulates microglial cell activation via the miRNA‐181a‐5p/SOCS2 pathway in vitro. A, Microglia activation was visualized by immunostaining with an anti‐Iba‐1 antibody in kainic acid (KA)‐activated BV‐2 microglial cells with control vector or circHivep2 overexpression plasmid, si‐NC or si‐ circHivep2 (n = 3/group). Scale bar = 50 μm. B, Protein expression level of Iba‐1 was determined by Western blot analysis in KA‐activated BV‐2 microglial cells (n = 3/group). C and D, The mRNA expression (C) and protein expression (D) of TNF‐α and IL‐1β were determined by RT‐qPCR and Western blotting in BV‐2 microglial cells in response to KA with or without circHivep2 knockdown or overexpression treatments (n = 3/group). E, Western blotting analysis of miR‐181a‐5p overexpression on the protein expression of Iba‐1, TNF‐α and IL‐1β, SOCS2 in KA‐activated BV‐2 microglia cells with control vector or circHivep2 overexpression plasmid (n = 3/group). The densities of the Iba1, TNF‐α and IL‐1β, SOCS2 bands were normalized to the β‐actin bands for each sample. Data are the means ± SD. *P < .05, **P < .01. Student's *t* test or two‐way ANOVA

To discern the effects of circHivep2 on KA‐induced activation of microglia, the morphological changes were evaluated by observing the level of Iba‐1, a specific microglial marker. Immunostaining using an anti‐Iba‐1 antibody was performed on BV‐2 microglial cells treated with KA and over or under‐expressing circHivep2 (Figure 4A,B). The activation of microglia was increased by KA compared with controls. Overexpression of circHivep2 reduced the level of Iba‐1 whereas down‐regulation increased the levels of Iba‐1. We also measured the levels of the pro‐inflammatory cytokines, TNF‐α and IL‐1β, in the same cells (Figure 4C,D). Similar results to those obtained with Iba‐1 were obtained with TNF‐α and IL‐1β. Overexpression of circHivep2 significantly reduced the elevated levels of TNF‐α and IL‐1β induced by KA, whereas under‐expression of circHivep2 resulted in the highest levels of TNF‐α and IL‐1β. Overexpression of miR‐181a‐5p was found to eliminate the inhibitory effect of circHivep2 on microglia cell activation and circHivep2 up‐regulated *SOCS2* expression, whereas miR‐181a‐5p inhibited the *SOCS2* expression increased by circHivep2 in KA‐activated BV‐2 microglial cells (Figure 4E).

### 3.4 Protective effect of circHivep2+ exosomes in mice with KA‐induced epileptic seizures

**Figure 5:**
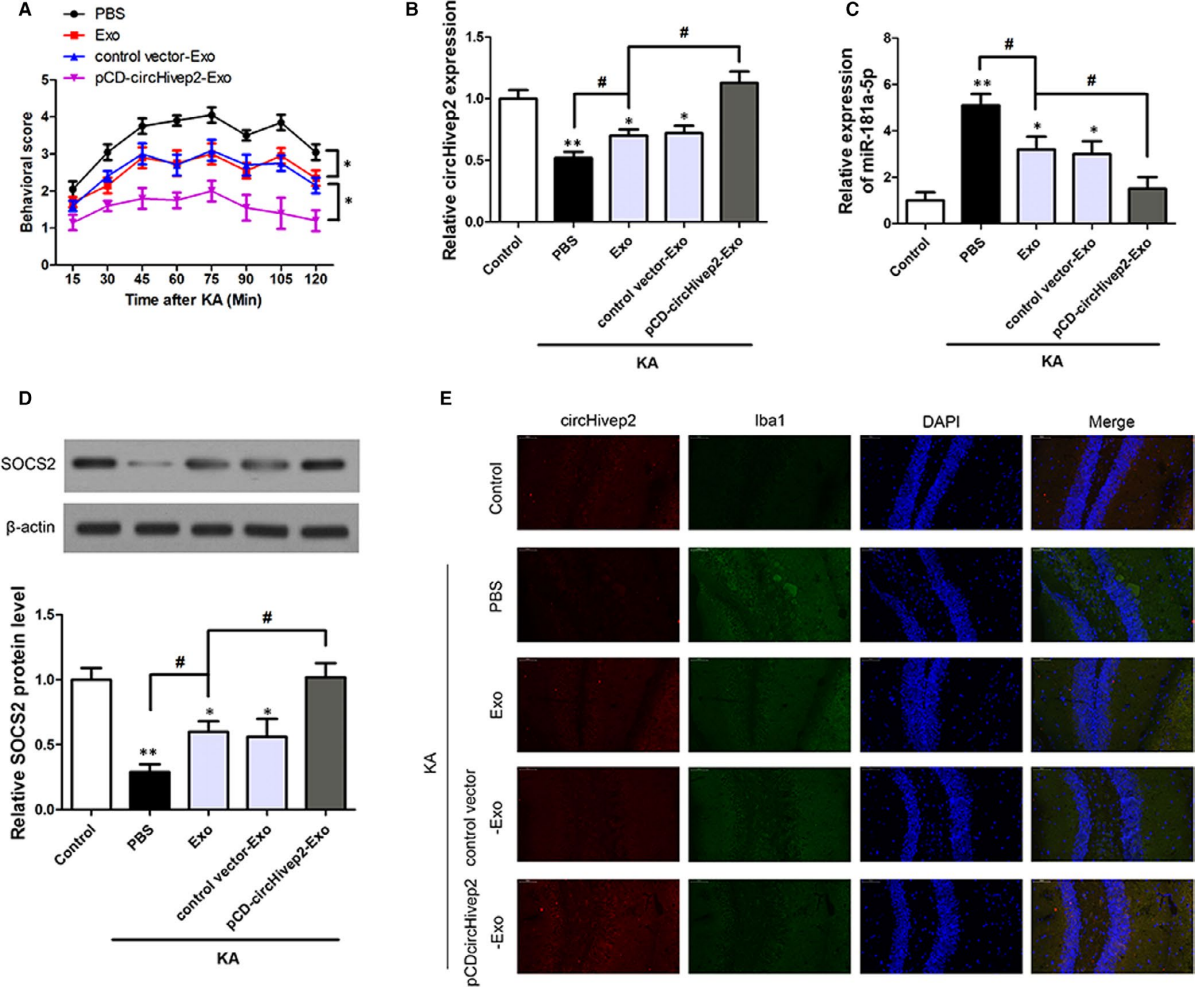
Effect of circHivep2+ exosomes on kainic acid (KA)‐induced seizures and on the expression of the miR‐181a‐5p/*SOCS2* pathway in epileptic mice. A, Behavioural scores were obtained over a time course of 120 min post–KA‐seizure induction to discriminate effects of circHivep2+ exosomes vs exosomes treatment compared to PBS‐treated mice with KA‐induced seizures(n = 10/group). *P < .05, vs Exo. B and C, RT‐qPCR verification of the expression of circHivep2 (B) and miR‐181a‐5p (C) in the hippocampus of mice KA‐induced seizures (n = 5/group). *P < .05, **P < .01, vs Control ^#^ *P* < .05, vs Exo‐KA. D, Western blot analysis of the protein expression of SOCS2 in the hippocampus of mice with KA‐induced seizures (n = 3‐4/group). The densities of the SOCS2 bands were normalized to the β‐actin bands for each sample. *P < .05, **P < .01, vs Control; ^#^ *P* < .05, vs Exo‐KA. E, Double‐labelling for circHivep2 (red) and Iba1 (green) in the hippocampal CA3 region of kainic acid (KA)‐induced mice with or without PBS, circHivep2+ exosomes or exosomes for 72 h (n = 3‐4/group). Scale bar = 50 μm

ADSCs are an important source of exosomes that can traverse the blood‐brain barrier and deliver microRNAs, drugs, proteins and other active agents to the brain. Therefore, we exploited this characteristic of ADSCs to overexpress circHivep2 and thereby acquire ADSCs‐derived circHivep2+ exosomes. The exosomes were ~100 nm in diameter, and positive for exosomal markers CD9, CD63 and TSG101 (Figure S2A,B). Compared with those in the control ADSC‐Exo, the levels of circHivep2 are significantly up‐regulated in the ADSCs‐derived circHivep2+ exosomes (Figure S2C). We used a KA‐induced mouse model of epileptic seizures to test the anticonvulsant effects of circHivep2+ exosomes. Behavioural scores were obtained over a time course of 120 minutes post–KA‐seizure. Compared with the PBS group, the injection of exosomes, control vector‐exosomes and circHivep2+ exosomes all exerted significant beneficial effects for the entire 120 minutes, and behavioural seizure scores were significantly decreased when compared to mice receiving an injection of exosomes not overexpressing circHivep2, suggesting that circHivep2 overexpression enhanced the protective effect of exosomes (Figure 5A). The expression of circHivep2 (Figure 5B) and miR‐181a‐5p (Figure 5C) was measured in the hippocampus of the KA‐treated mice. The expression of circHivep2 down‐regulated in PBS‐KA‐treated mice and increased in exosomes and circHivep2+ exosomes‐KA‐treated mice which contrary to the expression of miR‐181a‐5p, suggesting the negative regulation of circHivep2 on miR‐181a‐5p. Moreover, the expression of *SOCS2* increased with the increased expression of circHivep2, indicating that the suppression of miR‐181a‐5p could allow the expression of *SOCS2* (Figure 5D). To define the cell phenotype expressing circHivep2 in the hippocampal area, the expression of circHivep2 was determined by FISH in combination with immunohistochemical labelling for Iba‐1‐positive microglia. As shown in Figure 5E, circHivep2 expression was increased in the Iba1+ microglia cells in the hippocampus CA3 region in circHivep2+ exosomes‐KA‐treated mice, while circHivep2 was undetectable in PBS‐KA‐treated mice and the number of the Iba‐1+ microglia cells reduced after injection of circHivep2+ exosomes, suggesting that microglial activation could be repressed by overexpressing circHivep2 in mice with KA‐induced epileptic seizures.

**Figure 6:**
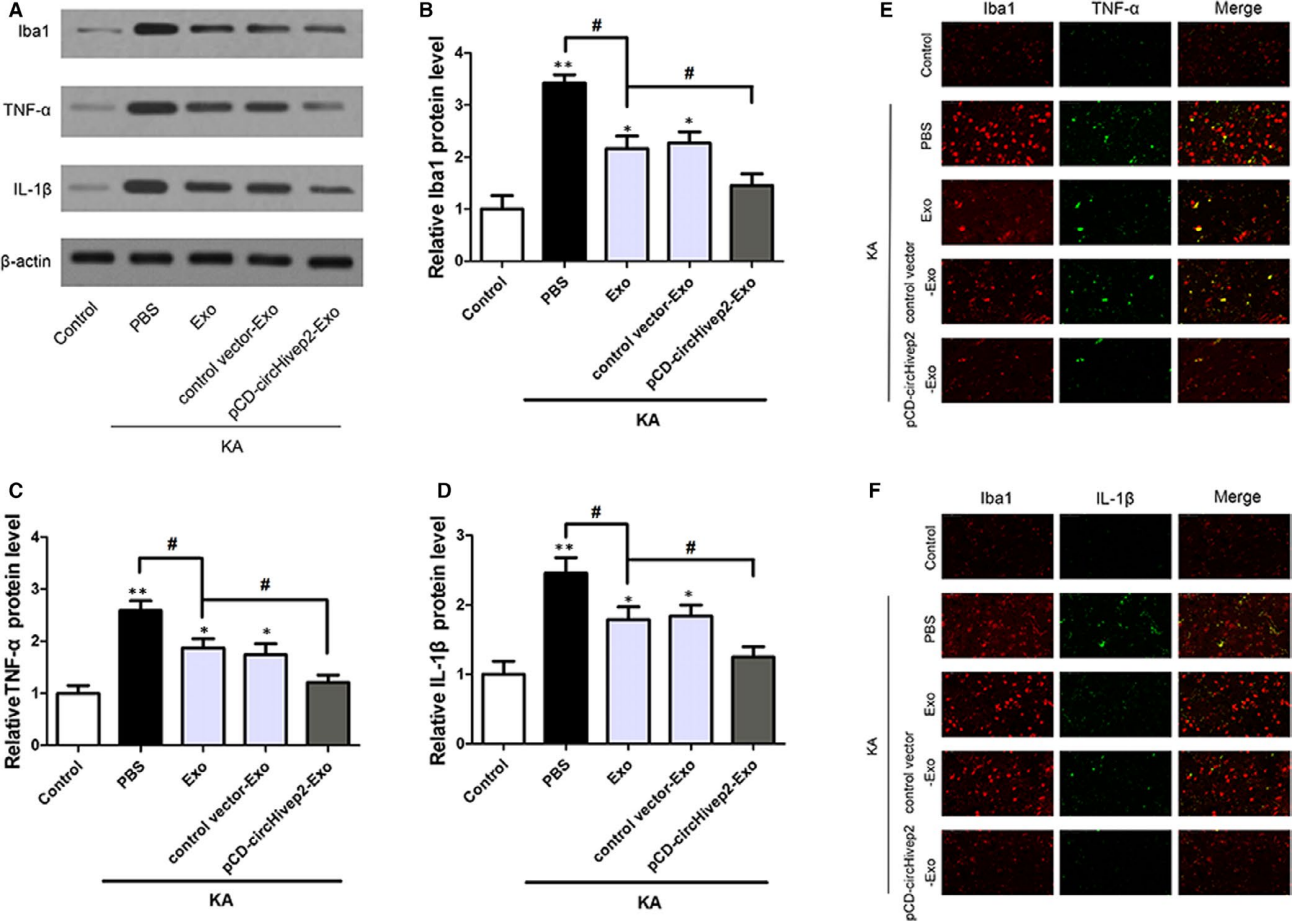
CircHivep2+ exosomes inhibited microglial activation and inflammatory response in the mouse model of epilepsy. A, Western blotting analysis of the protein expression of Iba‐1, TNF‐α and IL‐1β in hippocampus tissues of mice with KA‐induced epilepsy (n = 3‐4/group). Quantification of the protein expressions of Iba‐1 (B), TNF‐α (C) and IL‐1β (D). *P < .05, **P < .01, vs Control; ^#^ *P* < .05, vs Exo‐KA. Student's *t* test or two‐way ANOVA. E and F, Representative fluorescence micrographs of Iba1 (red) and TNF‐α (green), Iba1 (red) and IL‐1β (green) expression in the hippocampus of kainic acid (KA)‐induced mice with or without PBS, circHivep2+ exosomes or exosomes for 72 h. Scale bar = 50 μm. (n = 3/group)

To further understand the potential function of circHivep2+ exosomes in the hippocampus of mice with KA‐induced epileptic seizures, we examined Iba1 expression to evaluate microglia activation and the expression of pro‐inflammatory cytokines TNF‐α and IL‐1β, which may be released by activated microglia by Western blotting(Figure 6A‐D) and immunofluorescence (Figure 6E,F). As shown in Figure 6, the expression of Iba1 was markedly enhanced in the hippocampal at 72 hours after KA‐induced epilepsy. Microglial activation was significantly repressed after treatment with exosomes, and circHivep2+ exosomes further suppressed microglial activation compared to exosomes not overexpressing circHivep2. Furthermore, the exosomes significantly inhibited the release of TNF‐α and IL‐1β, and cytokine release was further decreased by circHivep2+ exosomes in the hippocampus at 72 hours after KA‐induced epilepsy. These results indicate that circHivep2 participates in the circHivep2/miRNA‐181a‐5p/*SOCS2* pathway to enable the regulation of epileptic seizures.

## 4 DISCUSSION

Epilepsy is a complex condition that affects 70 million people worldwide and is associated with several comorbidities and premature mortality.^45^, ^46^ Despite the increased development and availability of antiepileptic drugs, epilepsy is inadequately managed in a third of patients.^47^ Therefore, there is a need to develop more effective therapies to control and manage the disease. There is an increased interest in the use of miRNAs to achieve this objective.^48^ To this end, in the present study, we have contributed a dataset of circRNAs that are differentially expressed in hippocampus tissue during KA‐induced epileptic seizures in mice. The profile contains 1519 circRNAs, 627 of these were up‐regulated and 892 were down‐regulated. Of these differentially expressed circRNAs, we selected circHiveP2 for further investigation because it was consistently down‐regulated in mice with KA‐induced epileptic seizures. Moreover, HIVEP2 is related to neurological disorders in humans and is associated with numerous regulatory pathways involved in cellular immunity and development processes.^24^, ^27^, ^28^

The next step in our research was to establish the interactions and pathways associated with circHiveP2. We found through online queries that circHivep2 could interact with miR‐181a‐5p and verified this with a dual‐luciferase reporter assay. In humans, miR‐181a‐5p is associated with various cancers^38^ and is up‐regulated in multiple sclerosis.^37^ Mao et al^38^ discovered that miR‐181a‐5p interacts with fibroblast growth factor receptor 3 (FGFR3) to promote the progression of bladder cancer through the activation of the STAT3 pathway. Interestingly, both FGFR3 and STAT3 have been associated with epilepsy.^49^, ^50^, ^51^ FGFR3 mutations are associated with bilateral medial temporal lobe anomalies and focal epilepsy^50^ and inhibition of STAT3 was found to diminish seizures in pilocarpine‐induced SE.^49^ We further found that miR‐181a‐5p also targets *SOCS2*. SOCS proteins provide a negative feedback for cytokine interaction and have been previously implicated in epilepsy.^52^ Song et al^52^ discovered that Toll‐like receptor (TLR) 2 and TLR4 were significantly up‐regulated in a pentylenetetrazole‐induced rat model of epilepsy, which inhibited the expression of *SOCS1* and *SOCS3* and this, in turn, up‐regulated the expression of *STAT3*. SOCS proteins negatively regulate TLR‐mediated immune responses and can inhibit type I interferon (IFN) signalling.^53^ In the present study, we found that levels of Iba‐1, TNF‐α and IL‐1β were increased in KA‐induced epilepsy but overexpression of circHivep2 significantly reduced the elevated levels of TNF‐α and IL‐1β. Under‐expression of circHivep2 resulted in the highest levels of TNF‐α and IL‐1β. In contrast, overexpression of miR‐181a‐5p was found to eliminate the inhibitory effect of circHivep2 on microglial cell activation. Our results suggest that circHivep2 negatively regulates miR‐181a‐5p to prevent the up‐regulation of TNF‐α, IL‐1β and the activation of microglia cells. In addition, we found that overexpression of miR‐181a‐5p prevents the expression of SOCS2 leading to the activation of pro‐inflammatory pathways. KA induced the expression of miR‐181a‐5p but the presence of circHivep2 inhibited the induction of miR‐181a‐5p expression, indicating that circHivep2 could sequester miR‐181a‐5p or inhibit its expression. Studies have shown that TDP‐43 is pathologically accumulated in cytoplasmic aggregates in neurodegenerative disorders and regulates the activity of miRNAs biogenesis machinery, in particular, Drosha and Dicer.^39^, ^43^ We found that TDP‐43 could interact directly with circHivep2 and regulate biogenesis of miR‐181a‐5p, which suggests that circHivep2 could post‐transcriptionally regulate miR‐181a‐5p, through blocking the interaction between pri‐miR‐181a‐5p and TDP‐43.

Finally, we aimed to assess if circHivep2 could induce an anticonvulsant effect in the KA‐induced mouse model of epileptic seizures. Several studies have used ADSC‐derived exosomes to deliver miRNA into animal models of epilepsy.^31^, ^34^ Recently, the intravenous administration of ADSC‐derived exosomes was found to successfully deliver a miRNA across the blood‐brain barrier in a rat model of ischaemic stroke to inhibit the activation of microglia and the expression of inflammatory factors.^34^ In our study, microglial activation was significantly repressed after mice with KA‐induced epilepsy were treated with circHivep2+ exosomes. The levels of pro‐inflammatory cytokines, TNF‐α and IL‐1β, and cytokine release were further decreased by circHivep2+ exosomes in the hippocampus at 72 hours after KA‐induced epilepsy. These results indicate that circHivep2 may be part of the pathway circHivep2/miRNA‐181a‐5p/*SOCS2*, which may enable the regulation of epileptic seizures.

However, our study, while observing a real effect, has some limitations, given the small sample size and limited nature of the species studied. On the one hand, the experimental method has certain limitations, for example injecting the unlabelled exosomes. One study reported that 4 hours after intracerebroventricular injection, PKH26‐labelled EVs are able to cross the ependymal cell layer and penetrate the brain parenchyma. In our study, circHivep2 expression was increased in the Iba‐1+ microglia cells in the hippocampus area of exosomes and circHivep2+ exosomes‐KA‐treated mice, which indicated that the injected exosome should be able to reach the hippocampal area. However, the injected exosomes are not labelled and detected in the hippocampal area, this remains to be seen in further study. On the other hand, an anti‐inflammatory effect of circHivep2 treatment in mice with KA‐induced epileptic seizures has been observed only for a limited time span. Accumulating evidence from animal models has demonstrated that specific anti‐inflammatory treatments can be effective at both suppressing chronic seizures and interfering with the process of epileptogenesis.^54^ Blocking excessive inflammatory processes in the brain increases the seizure threshold and reduces the likelihood of recurrent seizures, thereby providing disease prevention or modification.^55^ However, the prevention effect of circHivep2 has been observed, and more experiments are needed evaluate the therapeutic effect of circHivep2 at chronic seizures. Third, the relevance of circHivep2 to granule cell dispersion (GCD) has not been rigorously studied. GCD is a characteristic structural abnormality in temporal lobe epilepsy.^56^ GCD is a process affecting differentiated granule cells, because neurogenesis is lost and replaced by gliogenesis in the dentate gyrus after intrahippocampal KA injection.^57^, ^58^ After unilateral intrahippocampal KA injection in adult mice, GCD develops and correlates temporally with a decrease of reelin expression.^58^ However, these histological changes to the dentate gyrus are a complicated process which is affected by various factors, little is known about the mechanisms that induce them. Hence, more experiments were needed to confirm the relevance of circHivep2 to GCD in our further study.

To conclude, the present study found that circHivep2 could inhibit neuroinflammation and the activation of microglial through the negative regulation of miRNA‐181a‐5p/*SOCS2* and thereby alleviate the symptoms of epileptic seizures in a mouse model.

## CONFLICTS OF INTERESTS

The authors declare that they have no competing interests.

## AUTHOR CONTRIBUTION

**Gao Xiaoying:** Data curation (equal); Formal analysis (equal); Investigation (equal); Methodology (equal); Writing‐original draft (equal). **Mian Guo:** Data curation (equal); Formal analysis (equal); Investigation (equal); Methodology (equal); Writing‐original draft (equal). **Liu Jie:** Data curation (equal); Formal analysis (equal); Investigation (equal); Methodology (equal); Writing‐original draft (equal). **Cui Ying:** Data curation (equal); Investigation (equal); Methodology (equal); Visualization (equal). **Zhu Yanmei:** Investigation (equal); Methodology (equal); Visualization (equal). **Shu Shengjie:** Investigation (equal); Software (equal); Visualization (equal). **Gou Haiyan:** Methodology (equal); Visualization (equal). **Sun Feixiang:** Investigation (equal); Software (equal). **Qi Sihua:** Conceptualization (equal); Funding acquisition (equal); Project administration (equal); Resources (equal); Supervision (equal); Validation (equal); Writing‐review & editing (equal). **Sun Jiahang:** Conceptualization (equal); Funding acquisition (equal); Project administration (equal); Resources (equal); Supervision (equal); Validation (equal); Writing‐review & editing (equal).

## Supporting information

Fig S1

Fig S2

Figure legends

## Data Availability

The data sets generated for this study are available on request to the corresponding author.
