# Supplementary figures and images for: CircHivep2 contributes to microglia activation and inflammation via miR‐181a‐5p/SOCS2 signalling in mice with kainic acid‐induced epileptic seizures
Source: J Cell Mol Med. 2020 Oct 1;24(22):12980–93. doi: 10.1111/jcmm.15894 (PMC7701587; doi:10.1111/jcmm.15894)

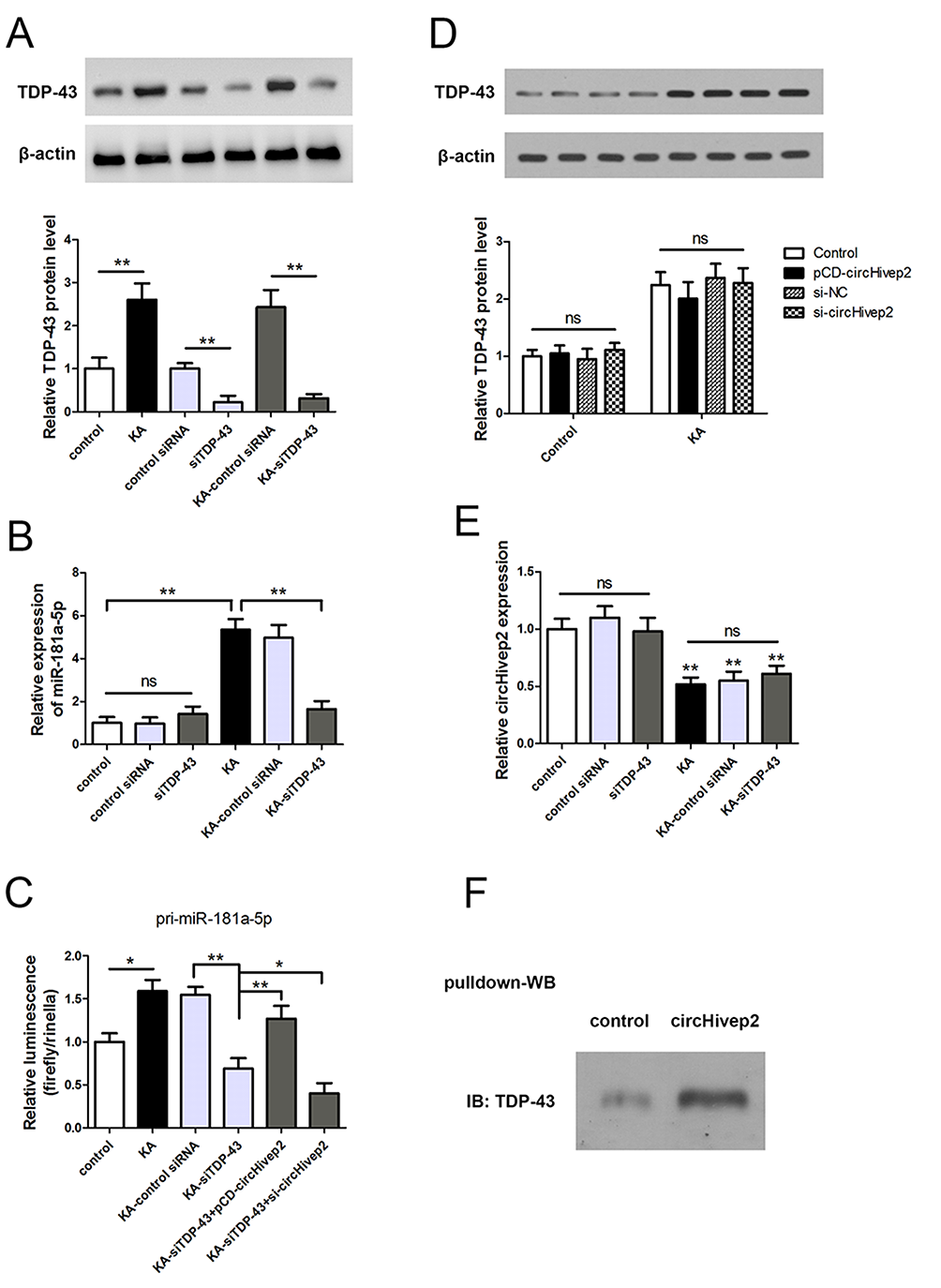

Supplement: Supplementary file 1 — Fig S1 [file JCMM-24-12980-s001.tif]

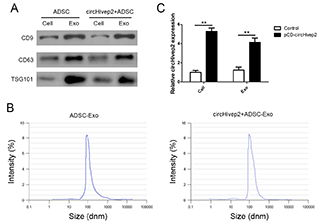

Supplement: Supplementary file 2 — Fig S2 [file JCMM-24-12980-s002.tif]
