# Supplementary material for: CircHivep2 contributes to microglia activation and inflammation via miR‐181a‐5p/SOCS2 signalling in mice with kainic acid‐induced epileptic seizures
Source: J Cell Mol Med. 2020 Oct 1;24(22):12980–93. doi: 10.1111/jcmm.15894 (PMC7701587; doi:10.1111/jcmm.15894)
Supplement: Supplementary file 3 — Figure legends [file JCMM-24-12980-s003.docx]

**Figure legends**

Figure S1 CircHivep2 and TDP-43 collaboratively regulate miR-181a-5p biogenesis. **A**, Western blot analysis of the protein expression of TDP-43 in BV-2 microglia cells with control siRNA or siTDP-43 after KA treatment (n=3/group). ** p < 0.01. **B**, The level of miR-181a-5p was determined by RT-PCR in BV-2 microglia cells with control siRNA or siTDP-43 after KA treatment (n=3/group). ** p < 0.01. ns, no significant. **C**, Decreased microprocessor processing of pri-miR-181a-5p as increased firefly/renilla luminescence in BV-2 microglia cells with control siRNA, siTDP-43 or/and circHivep2 overexpression plasmid after KA treatment (n=3/group). * p < 0.05, ** p < 0.01. Pri-miRNA processing assays were conducted as previously described by Jawaid A et. al (DOI: 10.1007/s12035-018-1314-3). **D**, Western blot analysis of the protein expression of TDP-43 in BV-2 microglial cells treated with control vector or circHivep2 overexpression plasmid, si-NC, or si- circHivep2 (n=3/group). ns, no significant. **E**, RT-qPCR for circHivep2 expression in BV-2 microglia cells with control siRNA or siTDP-43 after KA treatment (n=3/group). ** p < 0.01, vs. Control. ns, no significant. **F**, RNA pull-down followed by WB confirmed the interaction of circHivep2 with TDP-43 (n=3/group). Data are the means ± SD of three experiments. Student *t* test or two-way ANOVA.

Figure S2 Identification of ADSC-exosomes. **A**, Western blot analysis showed that exosomes (Exo) form ADSCs or circHivep2-overexpressed ADSCs expressed CD9, CD63, and TSG101 (n=3/group). **B**, Size distribution was measured by Nanosight, exosomes were ~100 nm in diameter (n=3/group). **C**, RT-qPCR for circHivep2 expression in control ADSCs or circHivep2-overexpressed ADSCs and their exosomes (n=3/group). ∗∗P < 0.01, vs. Control. Data are the means ± SD of three experiments. Student *t* test or two-way ANOVA.
